# Cecal metabolome fingerprint in a rat model of decompression sickness with neurological disorders

**DOI:** 10.1038/s41598-020-73033-z

**Published:** 2020-09-29

**Authors:** Sébastien de Maistre, Sandrine Gaillard, Jean-Charles Martin, Simone Richard, Alain Boussuges, Sarah Rives, Anne-Virginie Desruelle, Jean-Eric Blatteau, Catherine Tardivel, Jean-Jacques Risso, Nicolas Vallée

**Affiliations:** 1 Service de médecine Hyperbare Expertise plongée, Hôpital d’Instruction des Armées Sainte-Anne, BP 600, 83800 Toulon Cedex 9, France; 2 Université de Toulon, CS 60584, Toulon Cedex 9, France; 3 UMR INRA 12060/INSERM1263/AMU C2VN, Plateforme Métabolomique, Faculté de Médecine la Timone, 13385 Marseille Cedex, France; 4 Institut de Recherche Biomédicale des Armées-Equipe de Recherche Subaquatique Opérationnelle, 83800 Toulon Cedex 9, France

**Keywords:** Physiology, Diseases

## Abstract

Massive bubble formation after diving can lead to decompression sickness (DCS), which can result in neurological disorders. We demonstrated that hydrogen production from intestinal fermentation could exacerbate DCS in rats fed with a standard diet. The aim of this study is to identify a fecal metabolomic signature that may result from the effects of a provocative hyperbaric exposure. The fecal metabolome was studied in two groups of rats previously fed with maize or soy in order to account for diet effects. 64 animals, weighing 379.0_20.2 g on the day of the dive, were exposed to the hyperbaric protocol. The rats were separated into two groups: 32 fed with maize (Div MAIZE) and 32 fed with soy (Div SOY). Gut fermentation before the dive was estimated by measuring exhaled hydrogen. Following hyperbaric exposure, we assessed for signs of DCS. Blood was analyzed to assay inflammatory cytokines. Conventional and ChemRICH approaches helped the metabolomic interpretation of the cecal content. The effect of the diet is very marked at the metabolomic level, a little less in the blood tests, without this appearing strictly in the clinic status. Nevertheless, 37 of the 184 metabolites analyzed are linked to clinical status. 35 over-expressed compounds let suggest less intestinal absorption, possibly accompanied by an alteration of the gut microbial community, in DCS. The decrease in another metabolite suggests hepatic impairment. This spectral difference of the ceca metabolomes deserves to be studied in order to check if it corresponds to functional microbial particularities.

## Introduction

When diving, the gases breathed in through the regulator are dissolved in the body tissues progressively during the descent to the seabed. During the decompression phase they may give rise to the production of bubbles, even when there are no procedural faults. When bubbles form in excessive quantities in the blood and tissues, symptoms of decompression sickness (DCS) may appear^1^. Conventionally it is acknowledged that the quantity of venous bubbles has a positive correlation to the risk of DCS^2,3^. However, there is high intra- and inter-individual variability in terms of bubble formation for the same dive profile.

Neurological lesions with spinal cord and brain injury are the origin of the most serious and most frequently encountered symptoms in decompression sickness (DCS)^3^. Despite the reference treatment with hyperbaric oxygen therapy, 20 to 30% of patients have sequelae after medical treatment for neurological DCS^4^. The identification and control of new factors favoring DCS is therefore a major challenge.

This study focuses more especially on the products linked to the activity of the digestive system and its microbiota. The microbiota is mostly composed of strict anaerobic micro-organisms which produce by fermentation, from exogenous (undigested carbohydrates and proteins) and endogenous (mucopolysaccharides, cell debris or even enzymes) substrates, proteins and amino-acids, intermediate metabolites (succinate, lactate) and also terminal metabolites such as short chain fatty acids (acetic, propionic and butyric acids), ammonia and gases including hydrogen^5,6^. In diving, it has been shown that the digestive tract could have an influence on the occurrence of decompression sickness through the activity of the intestinal microbiota^7–12^. In fact, during dives using hydrogen as the diluent gas for O_2_, the metabolism of hydrogen by the native intestinal microbiota of pigs could protect against DCS, by the intermediary of a reduction in the body burden of H_2_^7^. Conversely, during a previous study we have shown that the bacterial fermentation of undigested sugars, during the dive, is accompanied by a greater incidence of DCS, including during dives using a diluent gas other than hydrogen^11,12^. A part of the hydrogen formed by fermentation in the intestine diffuses through the intestinal barrier into the entire body. This endogenous hydrogen could have a detrimental effect in DCS. It could contribute to a direct increase in the inert gas burden during hyperbaric exposure, and be excreted in the form of bubbles during the decompression phase. Less intuitively, increased fermentation over the long term with production of hydrogen before the dive would limit the risk of DCS due to the possible antioxidant and neuroprotective properties of the hydrogen^10^. The metabolic pathways for the hydrogen by the intestinal microbiota, and more generally, the fecal metabolome in the risk of DCS must, therefore, be specified.

In this context, we seek to understand the interactions between accident-provoking hyperbaric exposures and the cecal metabolome, thus involving the intestinal microbiote and the host organism. Insofar as intestinal activity varies as a function of diet, comparing the metabolome of rats fed identically would not have been sufficiently restrictive to isolate the influence of diet in the event of an accident. We have decided to expose two series of rats, previously fed with maize (cereal) or soy (legume), to accident-provoking protocols. These seeds are of different composition, with in particular different patterns of fermentability^13,14^, and they could therefore influence the metabolites distribution in the intestine ^15,16^^{Coward, 1972 #7327},^^17,18^. The fecal metabolome of rats was analyzed in order to detect the effect of diet initially, and therefore better spotlight by a statistical process a metabolomic signature which could result from the specific effects of the accident-provoking hyperbaric exposure.

We hypothesize that a fecal metabolome fingerprint linked to DCS could be identified.

## Equipment and methods

### Animals and ethical statement

All procedures involving experimental animals complied with European Union rules (Directive 2010/63/EU) and French law (Decree 2013/118). The Ethics Committee of the Institut de Recherche Biomédicale des Armées approved this study in 2016. Sprague–Dawley male rats (Harlan laboratory, France) were housed in an accredited animal care facility, at 22 ± 1 °C. They were kept in cages both during rest and during the experiments and maintained on a regular day (6:00 am–6:00 pm)/night (12 h) cycle. Before the beginning of the study, food (kibble from Harlan Laboratories, 18% protein) and water were provided ad libitum.

In agreement with our Ethics Committee, we took inspiration from the Swiss veterinary guide to establish a form for monitoring the welfare of the animals^19^. A dedicated observer was responsible for scoring (from 0 to 3) the stress and pain felt by each animal. 0 corresponds to a zero degree of stress and three is the maximum. A degree of stress of 3 in one case or a total score of 12 represents a criterion for stopping the procedure. The items refer to vocalization, licking, the presence of tears, aggression or withdrawn behavior, labored breathing, motor or locomotor disorders with paralysis for example. In this study, no score reached 12, and it was not necessary to resort to anticipated euthanasia.

At the end of the experimentation, the animals were anaesthetized by induction with isoflurane (Bellamont, firstly at 5% then 2%), in order to save time and minimize stress, then by intraperitoneal injection (1 ml syringe, Omnican, B. Braun, Melsungen, Germany) of a mixture of ketamine (Imalgene 1000, 100 mg/kg, AstraZeneca, London, UK), acepromazine (Calmivet, 1,65 mg/kg, Vétoquinol S.A., Lure, France) and xylazine (Rompun 2%, 16 mg/kg, Bayer HealthCare, KVP, Kiel, Germany).

### Batches and food

30 days before the hyperbaric exposure, the rats (300–325 g; 9–10 weeks old) were separated into two equal groups and fed with maize or soy, at a rate of 30 g per day. 64 animals, 32 fed with maize (MAIZE) and 32 fed with soy (SOY), weighing 379.0_20.2 g (median_interquartile) the day of the dive, were exposed to the hyperbaric protocol.

The animals were fed by our technician and the rats were identified by a code unknown to the staff in charge of the physical examination, then sorted according to their weight in order to balance the dive groups. The clinic was established by another staff and the coding was only revealed afterwards for processing the results.

### Exhaled hydrogen

Exhaled hydrogen was measured before the dive in order to evaluate gut fermentation. For all rats, the amount of H_2_ measured in exhaled air provides a measurement of the rate of H_2_ production resulting from the bacterial fermentation of carbohydrates in the gut (and hence diffusing throughout the body via the bloodstream). To measure H_2_ in exhaled air, we considered that the breathing rate in rats is constant over time, with a mean rate of 225 ml min^−1^. The literature provides a resting value of 27.27 ± 2.39 ml min^−1^ 100 g^−1^ (mean ± standard deviation) for Sprague–Dawley rats^20^. The weight of the rats we studied was 379.0_20.2 g (median_interquartile). However, taking into account the stress induced by the measurement process, we used a breathing rate double that of the value in the literature for unstressed, resting rats. For each measurement, each rat was placed in a clean, dry polyvinyl chloride (PVC) cylinder (internal diameter 75 mm, length 200 mm, i.e., an internal volume of 883 ml). Both ends were hermetically sealed using plastic discs with holes in the middle, allowing air in at one end and the collection of gases at the other end. Air was circulated by a special aerator (Rena Air 200, France) set to a constant flow rate of 225 ml ml min^−1^ which was controlled by the rise of a soap bubble in an inverted 100 ml test tube that was pierced at the bottom. After 5 min (to allow time for the gases to mix inside the cylinder, allowing for the dead space, i.e., that not occupied by the animal), successive measurements of H_2_ (ppm) in the air coming out of the cylinder were performed by means of a three-way tap. H_2_ was measured using a mobile exhaled H_2_ analyzer (Gastrolyser, Respur International, France). The results were recorded one hour before pressurization.

### Hyperbaric exposure

To remain comparable, we have graciously used the protocol of our previous experiences^9,10,12,13^. Batches of 8 freely-moving rats (4 per cage and 4 per group) were subjected to the hyperbaric protocol, which generates decompressions sickness, in a 200 L caisson with three observation portholes.

The protocol has two compression speeds. The animals were subjected to an air compression procedure at a speed of 10 kPa min^−1^ up to an absolute pressure of 200 kPa (corresponding to a depth of 10 m of seawater); and then a speed of 100 kPa min^−1^ up to a pressure of 1000 kPa (corresponding to a depth of 90 msw) where they remained for 45 min. The rats were then decompressed at a speed of 100 kPa min^−1^ up to 200 kPa, and then a speed of 10 kPa min^−1^ until return to normal pressure, adhering to 5-min stages at 200 kPa (10 msw) and 160 kPa (6 msw) with a final stage of 10 min at 130 kPa (3 msw). The decompression speed was automatically controlled by a computer connected to an Analogue/Digital converter (NIUSB-6211, National Instrument, USA), itself connected to a solenoid valve (Belimo LR24A-SR, Switzerland) and a pressure transmitter (Pressure Transmitter 8314, Bürkert Fluid Control Systems, Germany). The program used to control the compression and decompression speeds was devised by a laboratory engineer at DASYLab (DASYLab National Instruments, USA). The compressed air was supplied by a diving compressor (Mini-Verticus III, Bauer Comp Holding, Germany) coupled to a 100-L unit at 30 MPa, and connected to a pressure relief valve (LTHS 400 0086, ALPHAGAZ, Rousset, France). The oxygen analysis was performed using a micro-fuel electrochemical cell (G18007, Teledyne Electronic Technologies, Analytical Instruments, USA). The CO_2_ produced by the animals was captured with soda lime (< 300 ppm, GE Healthcare, Helsinki, Finland). The gases were mixed by a fan, and the temperature inside the caisson was measured with a heat probe (Pt 100, Eurotherm, France).

### Diagnoses and behavioral tests

The physical examination for the rats was conducted by the main experimenter. It was established over a 30-min observation period with the collection of clinical signs, where the respiratory difficulties, motor disorders, convulsions, and death were referenced with a time index. Then, these observations were completed by a Motor Performance Score (MPS), from 10 to 0, comprising specific tests for (loco)motor disorders ^21^: the *beam-walk test* from 1 to 7 (agility test on a 1.5 m long and wide plank calibrated from 7.7 cm to 1.7 cm, and placed 1.1 m above the void) was practiced two weeks before the dive and after the dive. It involved allowing the rat to move on an ever-narrower board above the void. The *rollover test* consisted of a simulated fall situation causing a reflex rollover in the animal so that it fell on its paws. It was assessed on a score of 0 to 2. The *toe-spreading reflex test*^22^ assessed motricity and, more especially, the functional impairment of the sciatic nerve (SFI Index). It was based visually on the spreading of the toes where 2 is a normal state, 1 a weak spread and 0 complete inability to spread the toes. This test was seconded by the diagnosis of motor impairment of the hind paws (MIHP), where a score of 5 indicates normal motricity, 4 a rat which limps, 3 a paw which is stretched and does not go back into place spontaneously, 2 a paw spontaneously to the rear, 1 a paw that no longer moves but is still capable of muscle contraction and 0 an inert paw.

### Clinical status

To remain comparable, we have graciously used the same protocol of our previous experiences^9,10,12,13^. The DCS status was attributed when the rat presented serious neurological signs in the form of paresis or paralysis of at least one limb, convulsions and/or reduced performance in SFI, MIHP locomotor tests, with a *beam walk test* score reduced by at least 2 points. The other rats were considered to be No DCS.

### Anesthesia and sacrifice

30 min after coming out of the hyperbaric chamber, all the animals were anesthetized by induction with isoflurane (Bellamont, firstly at 5% then 2%), then by intraperitoneal injection (1 ml syringe, Omnican, B. Braun, Melsungen, Germany) of a mixture of ketamine (Imalgene 1000, 100 mg/kg, AstraZeneca, London, UK), acepromazine (Calmivet, 1,65 mg/kg, Vétoquinol S.A., Lure, France) and xylazine (Rompun 2%, 16 mg/kg, Bayer HealthCare, KVP, Kiel, Germany).

At the end of the experiment, rats were sacrificed by an injection of sodium pentobarbital (200 mg/kg IP; Sanofi, Paris, France).

### Blood analyses

We have used our previously described protocol ^9,10,12,13^. Briefly, the blood counts were performed from 15 µl blood taken from the tip of the tail and diluted in the same volume of 2 mM EDTA (Sigma, France). The analysis was performed using an automaton (Scil Vet abc, SCIL Animal Care Company, France) on samples taken 60 min before or 30 min after exposure to the hyperbaric protocol. The values for the second blood sample were corrected depending on the variation in the hematocrit.

### Cytokine detection

We have graciously reproduced our analysis protocol^9,10,12,13^. Under anesthesia, blood samples were collected by an intra-aortic puncture to determine the values of plasmatic cytokine levels. Blood was collected in sterile 4 ml tubes containing lithium heparin (BD Vacutainer, BD-Plymouth, UK) and, within 30 min, plasma was separated out by simple centrifugation at 1200 *g* and 4 °C for 15 min. The supernatant was kept at − 80 °C until testing.

The pro-inflammatory cytokine IL-1β and oxidative stress markers TBARS and GPX were assayed using a rat ELISA kit (ELISA Kit, Antibodies-Online GmbH, Germany) and QuantiChrom TBARS Assay Kit and EnzyChrom Glutathione Peroxidase Assay Kit (BioAssay Systems, CA, USA). Samples, standards, and quality controls were all run in duplicate. All standards and quality controls were made up as recommended by the supplier.

### Fecal metabolome

Immediately before sacrifice, the cecum was separated from the rest of the digestive tract after ligation. The cecum was weighed and then opened using a scalpel. Part of its contents was placed in a 1.5-ml Eppendorf tube and kept at − 80 °C until metabolomic analysis.

100–150 mg of cecal content were homogenized in cooled methanol (3µL/mg feces) at − 20 °C. Samples were vortexed for 1 min and incubated at − 20 °C for 30 min. Samples were then centrifuged for 15 min (11,000 × *g*, 4 °C). The supernatant recovered from each sample was filtered through 10 KDa filter tubes by centrifuging for 45 min (11,000 × *g*, 4 °C). The extracts obtained were then dried using a stream of nitrogen and then frozen at − 80 °C.

LCMS metabolomic analyses were performed essentially as described earlier^23^. All the dried polar extracts were first reconstituted with 150 µl acetonitrile/water (50:50; v:v). The samples were separated using high performance liquid chromatography (UPLC) ultimate 3000 (Thermo Scientific), coupled to a high-resolution mass spectrometer (HRMS), Q-Exactive Plus quadrupole-orbitrap hybrid equipped with electrospray ionization source (H-ESI II). The chromatographic separation was performed on a binary solvent system using a HILIC column (Merk,SeQuant ZIC-HILIC, 150 mm × 2.1 mm, 5 μm, 200 A) at 25 °C with a flow rate of 0.25 ml min^−1^. The injection volume for both columns was 5 μl. The mobile phase for the HILIC column separation consisted of a combination of solvent A (100% water, 16 mM ammonium formate) and solvent B (100% acetonitrile 0.1% formic acid). The following gradient conditions were used: 0 to 2 min, isocratic 97% B;, 2 to 10 min, linear from 97 to 70% B; 10 to 15 min, linear 70 to 10% B; 15 to 17 min, isocratic 10% B; 17 to 18 min linear from 10 to 97% B; from 18 to 22 min isocratic 97% B. The separated molecules were analyzed in both positive and negative ionization modes in the same run. The repeatability of the analysis was checked by analyzing interspaced (1 out of every 5 samples) quality control samples (QC).

Data processing and molecule identification: All the raw data generated by the LCMS were converted to mzXML by ProteoWizard (Version 2.0), and then processed by MZmine 2.26. The identification of the metabolites was performed by using an in-house database referencing more than 800 metabolites with their chromatographic retention time acquired with a HILIC column, together with their exact mass and MSMS spectra obtained in positive and negative ionization modes, including their adducts and neutral losses. These led to Level 1 (MSMS, retention time, MS) or 2 identification (retention time, MS).

The MS metabolomics data from feces were merged into a single dataset together with the other corresponding biological data and microbiota measurements, giving rise to 185 variables per rat.

### Statistical analyses

The blood count analyses are calculated according to an individual variation percentage. For the groups the data is expressed in median and interquartile. Most series of values fell between 0 and 1 and the distribution was positively skewed. Prior analysis, scale-contracting transformation was applied with log(X + 1). The difference is analyzed using 2-way ANOVA (type III SS) on clinical status and diet, comprising interactions, followed by post-hoc Tukey’s (HSD) and Benjamini-Hochberg’s (False Rate Discovery) tests. Principal component analysis (Pearson correlation coefficient), agglomerative hierarchical clustering (AHC) (dissimilarity; Euclidean distance; Ward’s method) helped by k-means clustering were used to design the heat map and volcano plot, from normalized data of the 185 features of the 65 diving rats. The software was Xltat Biomed from Addinsoft. Maximum acceptable alpha level was 5%.

## Results

### Clinical observation

**Figure 1:**
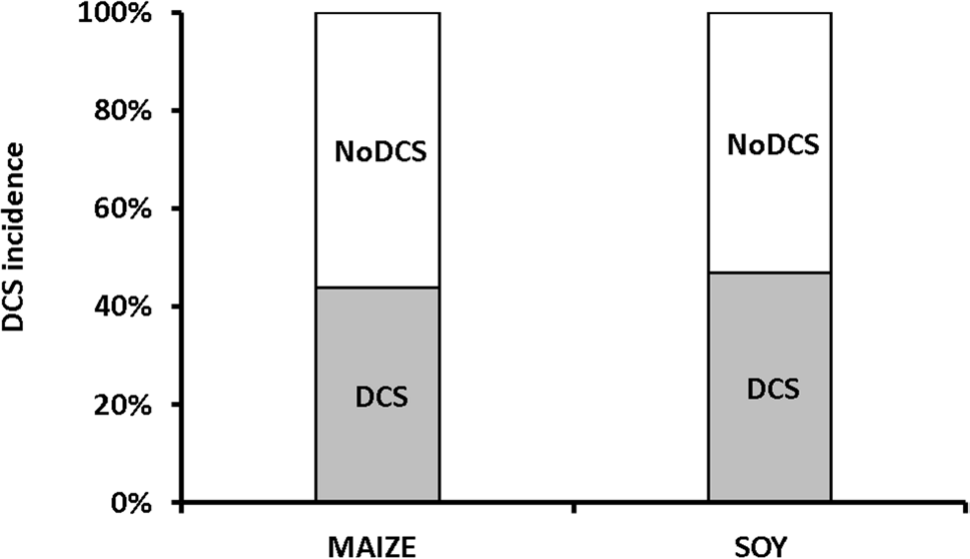
Percent of symptomatic rats 30 min after an at-risk decompression as a function of diet. Grey blocks were attributed to DCS status, i.e. when the rats presented neurological signs. White blocks represent the proportion of rats that shown no clinical sign (NoDCS).

The hyperbaric protocol did indeed generate decompression sickness. The incidence of clinical signs of neurological DCS (Fig. 1) is not significantly different between the groups of rats fed on maize and those fed on soy (n = 32/32, Levene median p = 0.806), with 44% and 47% of rats symptomatic respectively (Fig. 1). 8 and 7 rats succumbed to the sequelae of their DCS (MAIZE n = 8; SOY n = 7). Finally, there are no significant differences in any of the clinical examinations that are directly related to diet.

### Blood and exhaled hydrogen analysis

**Figure 2:**
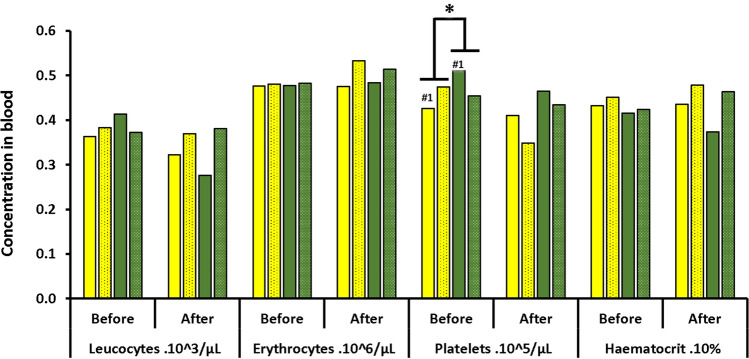
Blood cells count before and after the hyperbaric exposure. * notes significant difference (p < 0.05) for a whole diet group and # denotes difference between infra-groups. Yellow blocks represent the rats fed with maize. Green blocks represent the rats fed with Soy Blocks with dots indicate rats displaying symptoms of decompression sickness (DCS).

**Figure 3:**
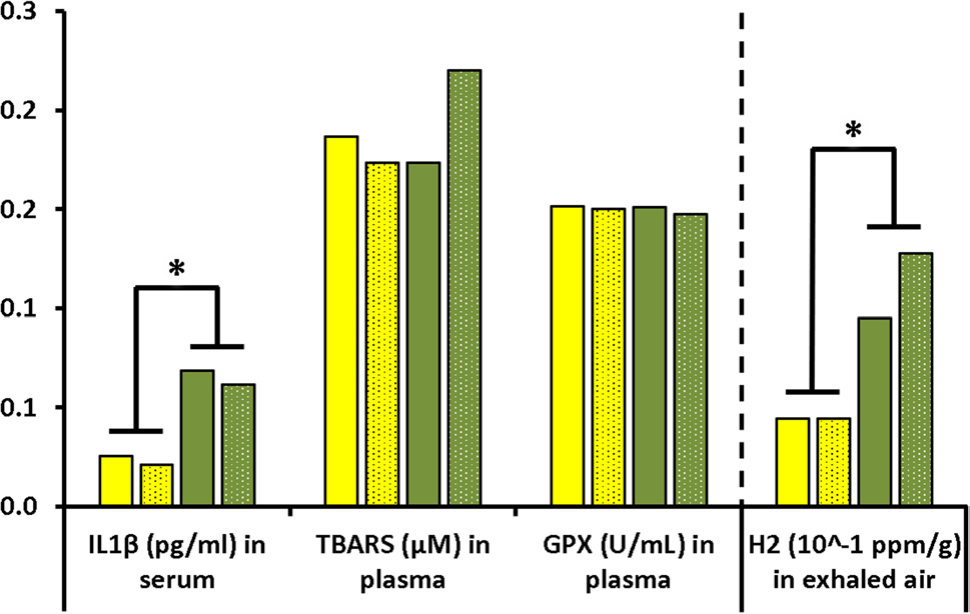
Concentrations of IL-1β TBars and GPX in blood and amount of hydrogen in exhaled air of rats submitted to a provocative decompression protocol. Yellow blocks represent the rats fed with maize. Green blocks represent the rats fed with Soy Blocks with dots indicate rats displaying symptoms of decompression sickness (DCS).

The diet modified a certain number of blood parameters in rats exposed to the hyperbaric protocol (Figs. 2 and 3).

The SOY rats had more platelets before the dive (median_interquartile. PLA_MAIZE_ = 450_80 × 10^3^/µl; PLA_SOY_ = 478_106 × 10^3^/µl; p = 0.022). This difference was more marked between the NoDCS MAIZE and SOY rats (p = 0.024). However, there does not appear to be any significant difference concerning their mobilization (p = 0.654). The red corpuscles were smaller in the SOY rats (MCV_MAIZE_ = 48.0_3.0 µm^3^; MCV_SOY_ = 44.0_5.3 µm^3^; p = 0.006) and this difference continued after the dive (MCV_MAIZE_ = 47.0_5.0 µm^3^; MCV_SOY_ = 43.0_7.2 µm^3^; p = 0.003). No other count difference (post-correction) linked to diet or clinical status was observed after the dive (Fig. 2).

The level of IL-1β is higher in SOY rats after diving (median ± interquartile IL-1β_MAIZE_ = 0.24_0.12 pg/ml; IL-1β_SOY_ = 0.68_0.16 pg/ml; p < 0.0001). No significance was noted for TBars and GPX (Fig. 3).

The level of exhaled hydrogen is higher in the SOY rats (median ± interquartile H2_MAIZE_ = 0.044_0.069 ppm/g; H2_SOY_ = 0.122_0.087 ppm/g; p = 0.001) and more specifically in the DCS rats (Fig. 3).

### Metabolomic analysis of feces

**Figure 4:**
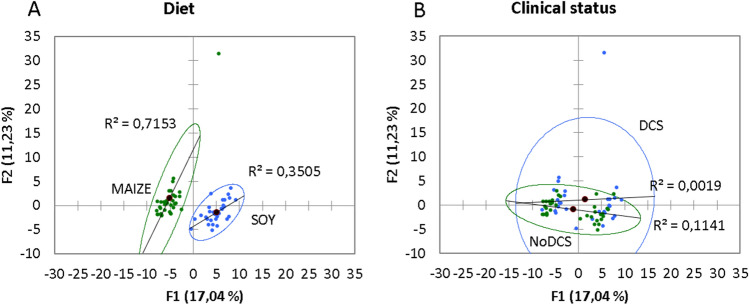
Principal component analysis (PCA) plot of fecal metabolome as a function of **(A) **diet (Soy and Maize) or **(B)** clinical status (DCS or NoDCS).

The principal component analysis (PCA; Pearson test) (Fig. 4) of the fecal metabolome of 64 rats highlights two distinct groups linked to diet, where the axes F1 and F2 explain 28% of the variability. A similar PCA conducted on the clinical status did not show any particular group.

**Figure 5:**
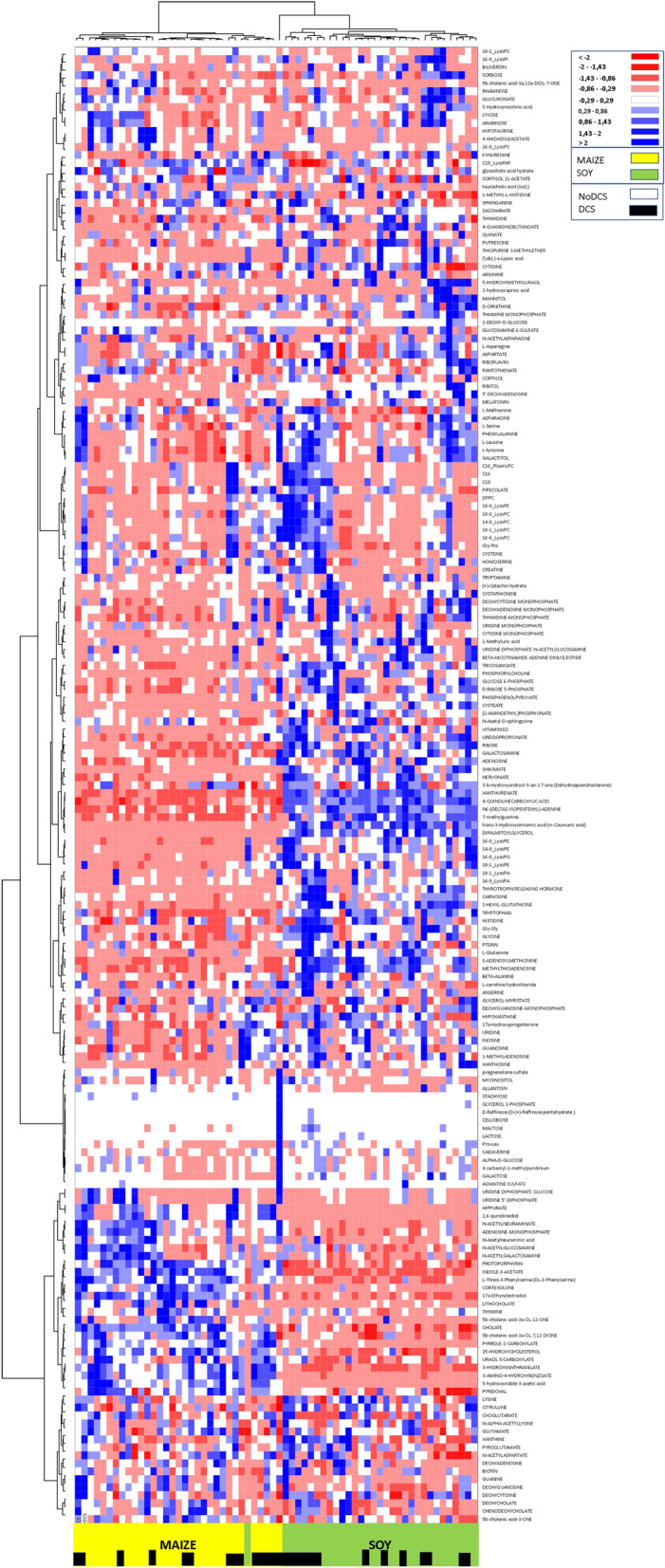
Heat Map, i.e. hierarchical clustering of fecal metabolomes from rats (n = 64) fed with maize (yellow) or soy (green). Black squares denote DCS status. Fold change for each metabolite is represented by a color. Intensity values are normalized, from red (Min: − 2) to blue (Max: + 2).

An Ascending Hierarchical Classification (AHC) was conducted in the context of the Heat Map established according to the intensity of the 184 compounds analyzed (Fig. 5). Following the evolution of the variances according to the number of classes, there is an inflection point in the 3rd class. This division explains 67% of the intragroup variability. Even though this distribution highlights 3 groups, 2 actually appear. Effectively the test spotlights 3 groups of rats (sorted according to the values of the compounds) but one group contained just a single rat, and was itself was situated between the 2 other groups. This means there were only 2 groups remaining, composed solely of either MAIZE or SOY rats. The effect of the diet is actually very marked. Finally, the distribution into 2 groups explains 86% of the intra-class variance. Therefore, the ACH of the Heat Map makes it possible to see distinctly on the abscissa a separation of the diets with rats fed on maize on the left (yellow) and those fed with soy on the right (green). An over-expression of the compounds in the feces of rats fed on soy is therefore visible in blue on the section at mid-height to the right of the map. The maize diet seems to favor an over-expression of the compounds situated in the bottom left of the map.

In addition, a more discreet dichotomy appears between the DCS and NoDCS rats, with a concentration at the center of the DCS rats (shown by black squares on the abscissa) whether they are MAIZE or SOY. It can be noted by vertical projection that for two thirds of the map there is a panel of molecules for which expression seems to be linked to clinical status.

**Figure 6:**
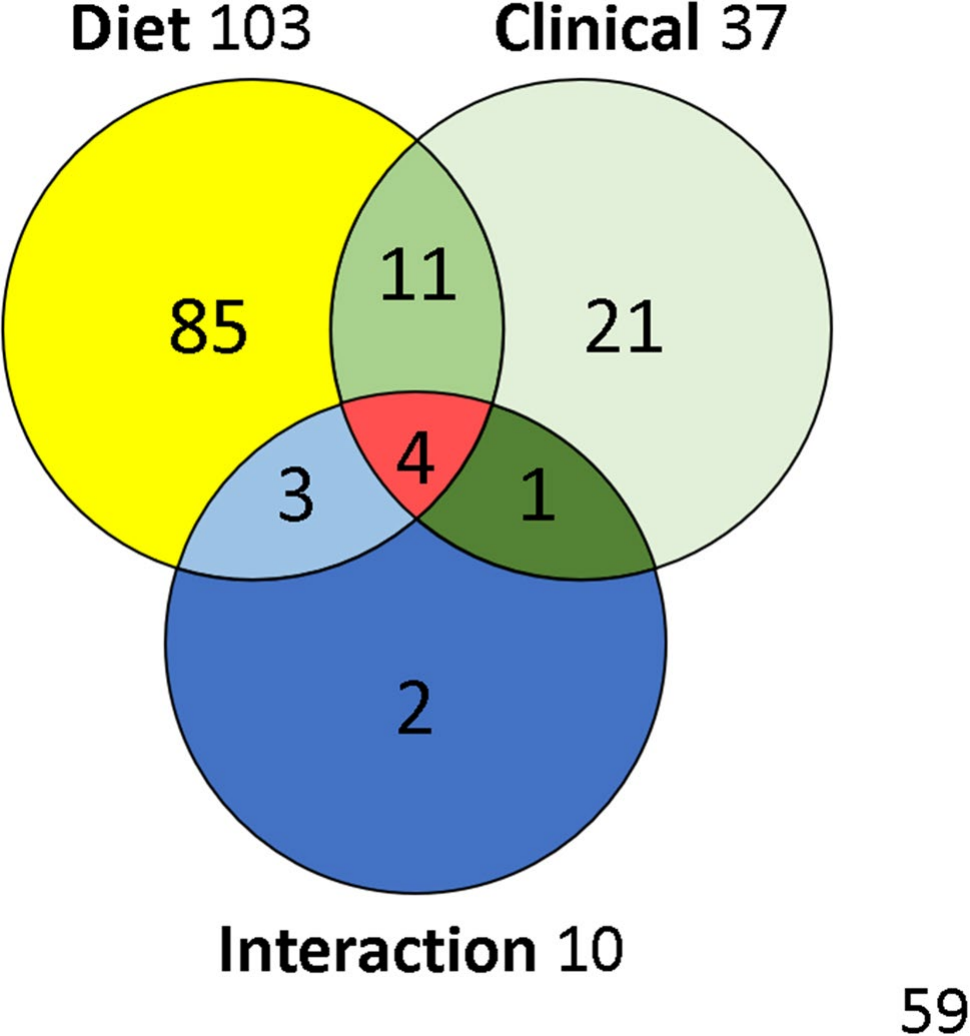
Venn diagram with metabolites influenced by diet or clinical status, with interactions. This graph is composed using 2-way ANOVA results.

The ANOVA (Type III SS with post-hoc Tukey (HSD) and Benjamini-Hochberg (FRD)) with two factors (Clinical status x Diet) has been performed for the 185 compounds in the 65 diving rats. The Venn diagram (Fig. 6 + Supplementary data 1 for the list of metabolites) from this analysis enables the effects linked to clinical status or diet to be seen, as well as their interactions. Out of the 185 compounds analyzed, 103 were significantly influenced by diet and 37 differed with clinical status.

**Figure 7:**
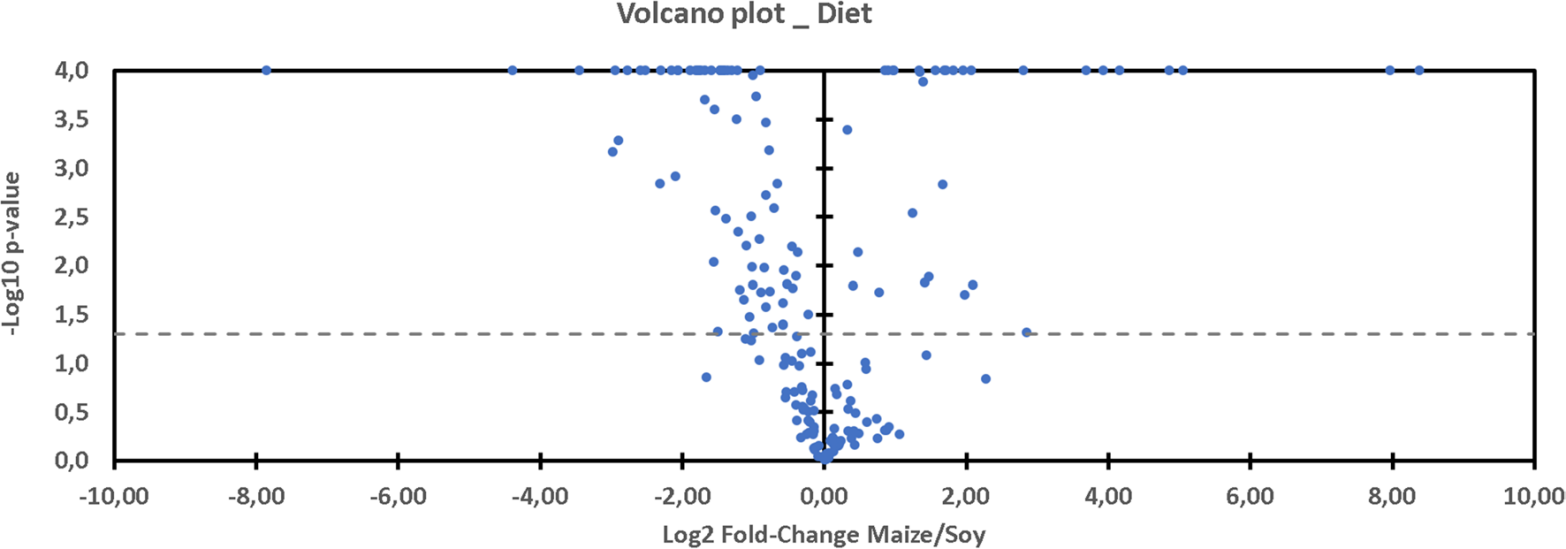
Volcano plot showing metabolomic data. Differential expression of fecal metabolites (n = 185) in rats exposed to the hyperbaric protocol (n = 64) favored by maize (right) or soy (left). The dashed line shows where p = 0.05 with points above the line having p < 0.05 and points below the line having p > 0.05.

It can be observed on Graph 7 (Volcano Plot, Fig. 7) that the expression in the rat feces of a large part of the metabolites is favored by the soy diet (71 versus 32) compared with the maize-based regime. It is therefore noted that the soy diet has a tendency to increase the quantity of amino-acids and saturated lysophospholipids, and to reduce that of cholic acids in the feces. As the study focuses on DCS, the details of the effects of the diet are not discussed here.

**Figure 8:**
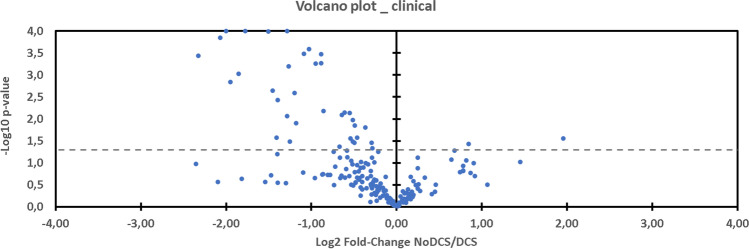
Volcano plot showing metabolomic data. Differential expression of fecal metabolites (n = 185) in rats exposed to the hyperbaric protocol (n = 64) favored by DCS (left) compared to NoDCS (right). The dashed line shows where p = 0.05 with points above the line having p < 0.05 and points below the line having p > 0.05.

In the case of diagnosis of decompression sickness, 35 metabolites are over-expressed and 2 under-expressed compared with those present in the feces of healthy rats (Volcano Plot, Fig. 8).

Amongst the metabolites linked to clinical status (Fig. 6), 11 are common to diet without there being any interaction (18-0_LysoPC, 18-0_LysoPE, 18-1_LysoPE, C18, carnosine, deoxyadenosine monophosphate, glycine, histidine, lithocholate, phosphorylcholine, tricosanoate). On the other hand, 4 other common compounds experience the effects of the interaction of 2 significant factors (dipalmitoylglycerol, hippurate, 2,6-quinolinediol, Gly-Gly). These 4 metabolites are all increased in the case of DCS, even though soy increases the expression of dipalmitoylglycerol and Gly-Gly, and decreases the expression of hippurate and of 2,6-quinolinediol. 2 other compounds would be modified (p = 0.025 and p = 0.040) by the joint action of diet and clinical status, without there being any pure effect due to diet alone or clinical status (glycerol-myristate p = 0.156, N-acetylasparagine p = 0.192), which indicates an antagonist effect of the 2 factors. 1 compound (kynurenine) is increased in the case of DCS (p = 0.027), and this effect is potentialized by the maize diet whereas it is not with soy (p = 0.043). Finally, 3 other compounds modified by diet (5b-cholanic acid-3a-OL-12-ONE p = 0.001, 5′-deoxyadenosine p = 0.0001, pyridoxal p < 0.0001) see their expression changed in the case of DCS (p = 0.037, p = 0.042, p = 0.034) but they do not seem to exercise determinism on the genesis of DCS.

## Discussion

### Impact of the hyperbaric protocol

The protocol has caused decompression sickness with neurological clinical signs. As expected, diet per se is not enough to induce DCS but we have been able to establish a link between the expression of fecal metabolites and the illness.

### Effects of diet

The purpose of this study is not to analyze and discuss exhaustively the influence of a diet on the different variables studied, but to identify the points influenced by these two diets in order to focus better on those which vary in the case of DCS. So we made the choice to describe only briefly the effects linked to food.

We have been able to determine that diet has influenced initial blood parameters Thus, the SOY rats have more platelets but their mobilization does not seem to be more affected. It is also noted that the volume of their erythrocytes is lower^24^. Amongst other things, this would tend to indicate that they are older or that their pool is renewed less frequently. However, the volume is not changed after the dive. Amongst the causes of microcytosis are inflammation (see below) and iron deficiencies^24^, but soy is reputed to be richer in iron, compared to maize^18^. In addition, these two parameters do not seem to determine the occurrence of DCS.

At the same time, the levels of exhaled hydrogen are higher in SOY rats, particularly in the DCS SOY rats, which could be a sign of more significant intestinal fermentation in these subjects. This result could be expected given the fermentable nature of soy. During a previous study we have already mentioned that the endogenous production of hydrogen by bacterial fermentation could have a beneficial effect over the long term, arguing for its neuroprotective effects^10^, or conversely harmful in the short term due to its participation in the expansion of bubbles^11^. The absence of clinical difference in this study does not favor any of these scenarios in particular, so this suggests an equilibrium related to their antagonism. An alternative explanation would be to consider hydrogen as an indicator of a susceptibility to DCS, without necessarily being a cause.

Although not significant as far as clinical status is concerned, the measurement of IL-1β is higher in the SOY rats after diving, which indicates the existence of inflammation.

As far as the fecal metabolome is concerned, as expected its composition depends on diet. So 103 out of the 185 metabolites identified vary as a function of diet (Suppl data 1). As the purpose of our study was principally to understand better the mechanisms of decompression sickness, using two different diets has enabled us to spotlight the metabolites which seem to be an indication for DCS. However, it goes without saying that these metabolites could not exist without food, and that by nature they are therefore influenced by diet and also by microbial activity. Finally, this method has enabled us to restrict our analysis to 37 metabolites.

### Metabolome in DCS

Out of the 37 metabolites linked to clinical status, about fifteen are common to the diet, including 4 which are modulated synergistically.

### Expression of metabolites indicating an alteration of the host

**Figure 9:**
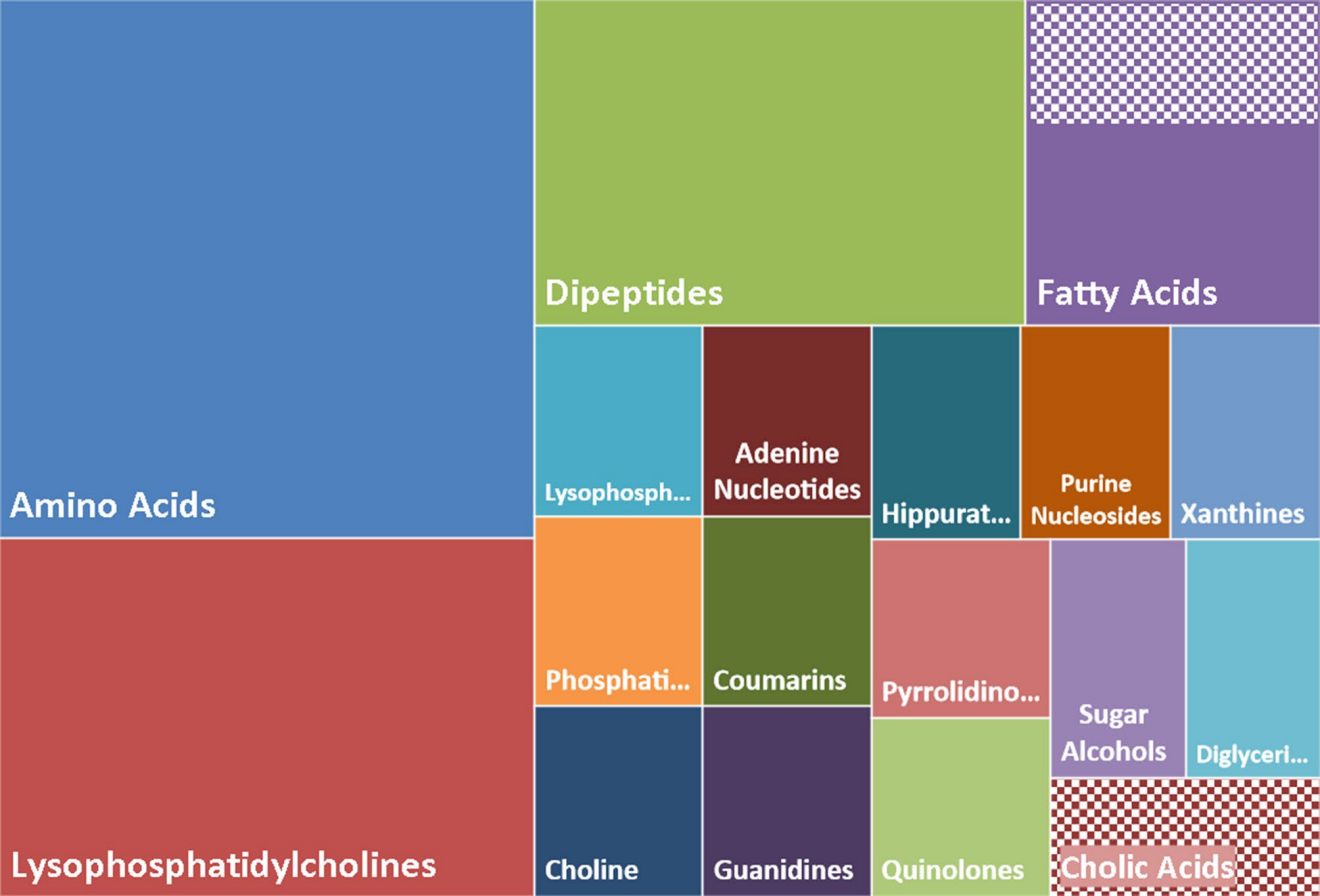
Clustering by chemical and biological similarities of the 37 metabolites significantly altered as a function of clinical status (ref. Venn diagram). The surface of the rectangles takes into account the number of metabolites. A chemical compound is 1 unit and the total area is 37 units. The striped areas correspond to an under-expression and the solid areas to over-expression.

The identification of the metabolites incriminated in DCS accentuates several large chemical families (Fig. 9) where, in animals suffering from DCS, an over-representation of amino-acids (9/37) and dipeptides (5/37), lysophosphatidylcholines (6/37) and other phospholipidic derivatives (2/37) is found. This excess basically suggests less degradation and also less absorption by the intestine. Furthermore, noted in these animals is decreased expression (checkers in Fig. 9) of a fatty acid (2-hydroxycaproic acid) and particularly cholic acid, lithocholate, which is one of the biliary acids, the function of which is to help absorb dietary fats by the formation of micellae. Their second role is to inhibit (by an antiseptic or even antibiotic effect) the proliferation of the bacteria in the upper part of the digestive tract. However, a larger role is now attributed to biliary salts throughout the body^25^. Coumarins are also found, which are not very soluble and also require a vector to be absorbed. This coumarin, C18 or 7-[(3-chlorobenzyl)oxy]-4-[(methylamino)methyl]-2 h-chromen-2-one, is an inhibitor of monoamine oxidase-B, which is abundant in the muscular layers of the rats’ colon. Its administration increases dopamine which inhibits colon motility^26^, which suggest mobility of the colon in DCS. This deserves to be confirmed.

This observation poses the problem of the integrity of digestive function in the context of DCS. As it happens, liver disorders have already been mentioned in DCS in humans^27,28^ and animals^19,29,30^.

### Expression of metabolites indicating an alteration in the intestinal microbiota

An increase in hippurate is observed in the DCS rat group, and more especially in the MAIZE DCS. In rats and also humans an increased quantity of this metabolite is regularly associated with a greater bacterial diversity in the microbiota^31–33^ even though it is also described as the marker for the abundance of bacteroides, the most important group of anaerobic Gram-negative bacilli in the intestine^34^. At the same time there is an excess of phosphorylcholine (choline), a molecule which in vaccination strategies^35,36^ activates the immune system. Phosphorylcholine is contained in all Gram-positive and Gram-negative bacteria, and its detection by the M cells (microfold cells) of the cecum, during bacterial over-representation or when there is a digestive tract lesion allowing infiltration, could participate in the inflammatory syndrome that is regularly described in DCS. More globally, the over-expression of this choline suggests that the bacterial populations have been affected. Another interesting marker of the microbiota that could corroborate this alteration of these populations is creatine which is normally excreted from the host by the action of the intestinal microbiota^37^. In this study the fecal levels of creatine were raised in the DCS rats compared with the NoDCS rats. Studies show an increase of these molecules in the biofluids of mice treated with antibiotics^25,38^ and in Germ Free mice^39^. As for the reduction in 2-hydroxycaproic acid, it could also signify a decrease in the activity of the anaerobic microbiota: its increase has been shown in buccal microbial community rich in anaerobic germs^40^. It is therefore worth investigating whether the accident-provoking protocol alters the expression of this metabolite by the microbiota as antibiotics do. A similar action could be expected by the high levels of oxygen (max inhaled PiO2: 2000 mbars) caused by the hyperbaric protocol on the essentially anaerobic bacteria.

Furthermore, xanthine, the pyroglutamate, derivatives of nucleotides and nucleosides, myoinositol, and the diglyceride (dipalmitoylglycerol) are over-represented.

### Expression of metabolites suggesting inflammation

Although it is speculative, it seems that in all the animals suffering DCS a reorganization of the activity of the intestinal microbiota emerges following a change in diet, which has moved from a standard diet to one based on soy or maize. This generally involves the overgrowth of bacterial populations (increased phosphorylcholine) which are trying to adapt to the new diet. This regularly involves an inflammatory reaction on the part of the host^41^. In the MAIZE DCS rats, this inflammatory response can be the consequence of a reorganization of the microbiota as is also suggested for the increase in hippurate^31–34^. As it happens, inflammation in the MAIZE DCS rats is suggested by the joint increase in kynurenine from the tryptophan metabolism^42,43^ and in 2,6 quinolinediol with antibiotic properties, which is thought to control an explosive development of the microbial community. It seems to be limited to the intestine^44^. Conversely in the SOY DCS rats, it would seem that the inflammatory mechanism differs where there is only an increase in the IL-1β in the blood.

Finally, it seems that the inflammatory phenomenon, wherever it comes from, is detrimental if there is exposure to an at-risk decompression protocol.

### Metabolomic analysis by ChemRICH

We also decided to submit our results to the ChemRICH database^45^ in order to access enrichment statistics and also to take into account differential expression rates (https://www.chemrich.fiehnlab.ucdavis.edu), and in this way compare our interpretations from the bibliography. It was possible to propose 185 compounds accompanied by the p-values and their variation factor. 183 compounds were accepted. This approach was conducted to analyze the effects of the diets (Supplementary data 2 with Fig. 11) and those linked to clinical status.

**Figure 10:**
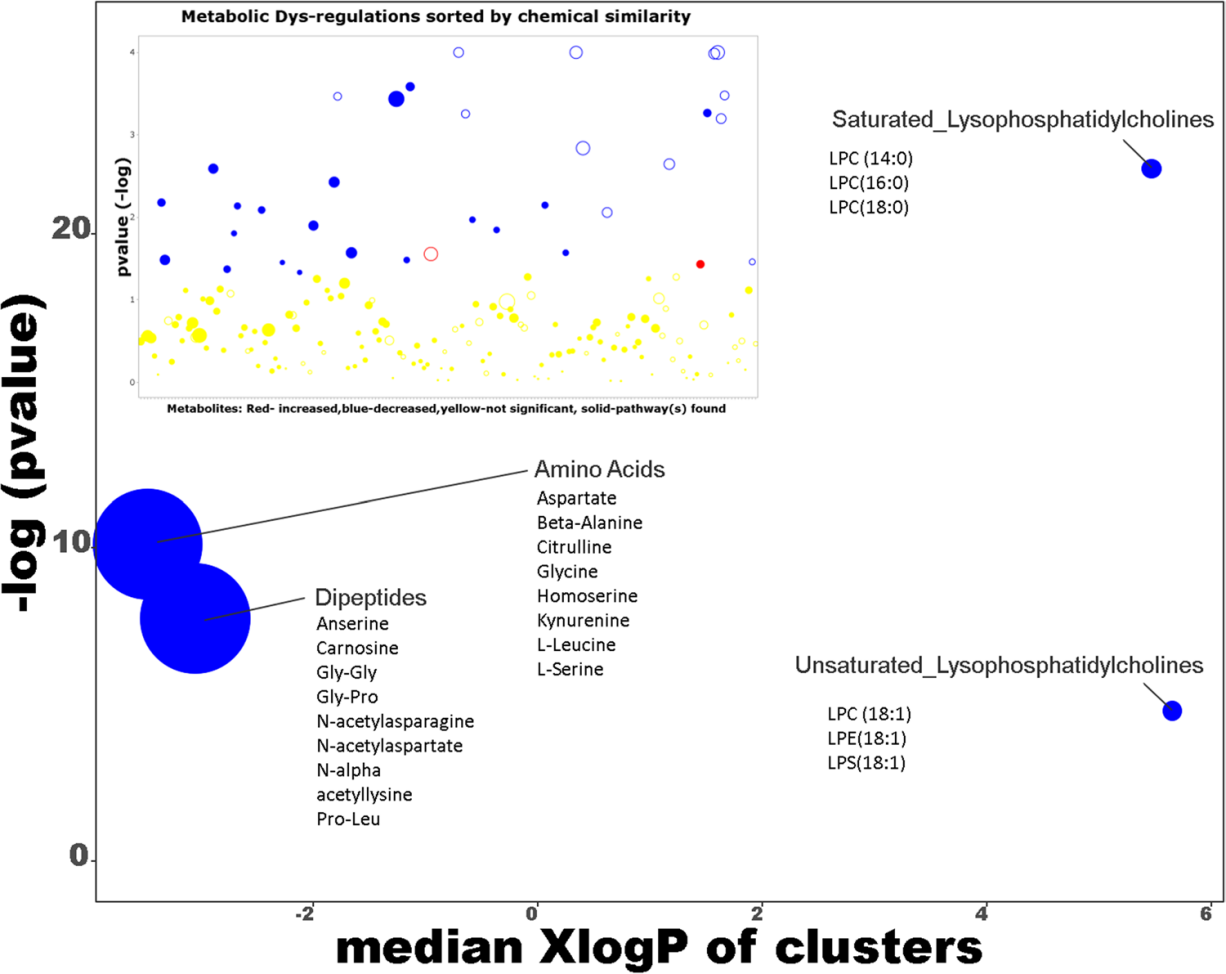
ChemRICH set enrichment statistics diagram. Each disc reflects a significantly altered family of metabolites. Enrichment p-values are given by the Kolmogorov–Smirnov test. Disc sizes represent the total number of metabolites in each group set. Red discs present Increased metabolites while blue ones show decreased compounds in NoDCS rats compared to DCS rats. Intermediates color have both increased and decreased metabolites. For example, there are fewer amino-acids in the feces of NoDCS rats, which is to say that there are more in DCS rats. Insert: volcano plot showing the metabolic dys-regulation in rats exposed to a provocative dive, detailing which of the most significantly altered metabolites were not mapped to metabolic pathways.

The graph from the ChemRICH database (Fig. 10), which uses their chemical similarity to group together the metabolites that are significantly altered depending on clinical status, makes it possible to note a general increase in the quantity of amino-acids, dipeptides, and also saturated and unsaturated lysophosphatidylcholines in the feces of rats affected by DCS.

As regards the excess of dipeptide in the feces, these results seem to show less protein breakdown in the intestine with, at the same time, less absorption of amino-acids. On this graph a larger amount of lysophosphatidylcholine, and constituents of the bacterial membranes is seen.

This result agrees with the previous result even though the ChemRICH analysis only shows 22 compounds, including only 12 of the 37 metabolites deemed to be significantly altered by ANOVA-2.

### Moderation and openness

It must be remembered that this work has not studied cecal composition exhaustively and that it is possible that metabolites which were not analyzed in this study may play a determinant role.

We have shown that a certain number of cecal metabolites are linked to DCS without making a direct link between diet and diagnosis. However, as this study was linked to maize and soy, it is not possible to exclude the fact that another diet could have an influence on a large number of compounds linked to clinical status and thus actually induce DCS. Conversely, it would seem interesting to determine a diet, or even modulate the intestinal microbiota, with the purpose of targeting this range of metabolites and thus seek to reduce the impact of the hyperbaric protocol.

We have mentioned less absorption and possible liver failure on the basis of the identification of specific metabolites, but it seems necessary to specify that the metabolites measured here are the fruit of digestion of exogenous (nutrition) and endogenous (intestinal cell debris, etc.) substrates, which are themselves subject to microbial activity (fermentation), and that as such it remains difficult to identify their exact source correctly. The identification of the bacterial sources present would probably make it possible to clarify this point. Nevertheless, a complete study would be necessary to discern the effects of diving and hyperoxia on the various bacterial strains (strictly anaerobic or not) and their RNA integrity, while knowing which falls under the diet. This proposal involves considerably increasing the number of animals required to know the situation before diving.

Finally, from a clinical point of view, it would be interesting to consider how much the alteration of the bacterial community affects the prognosis in the short and medium term, and consequently whether it too should be the subject of investigation. As a result, it should be taken into account in DCS therapeutic in order to improve its condition or avoid further degrading it, by limiting the side effects of hyperbaric oxygen therapy on anerobic species for example.

## Conclusion

This study shows for the first time the spectral fingerprint of the ceca metabolome in animals exposed to a provocative decompression, while evaluating the diet effects. On the one hand, statistical analysis shows a general over-expression (35/37 metabolites) of amino-acids, dipeptides, and lysophosphatidylcholines, and on the other a reduction in lithocholate, a biliary salt in animals suffering of decompression sickness. These results suggest an intestinal disorder with alteration in the microbial community with the liver being affected, probably linked to inflammation, but additional studies must verify this more specifically.

More generally, we have demonstrated that the choice of a diet, insofar as it modifies the fecal metabolome, may favor the over-expression of some metabolites linked to the pathology. Even though the diet alone may not be enough to induce decompression sickness, it would seem prudent to verify, in future studies, whether other diets can limit this expression and thus reduce the impact of hyperbaric exposures.

## Supplementary information

Supplementary Information.
