# Supplementary material for: Cecal metabolome fingerprint in a rat model of decompression sickness with neurological disorders
Source: Sci Rep. 2020 Sep 29;10:15996. doi: 10.1038/s41598-020-73033-z (PMC7524739; doi:10.1038/s41598-020-73033-z)
Supplement: Supplementary file 1 — Supplementary Information. [file 41598_2020_73033_MOESM1_ESM.docx]

**Supplementary Data**

Cecal Dysbiosis in a rat model of decompression sickness with neurological disorders.

Sebastien De Maistre, Sandrine Gaillard, Jean-Charles Martin, Simone Richard, Alain Boussuges, Sarah Rives, Anne-Virginie Desruelle, Jean-Eric Blatteau, Catherine Tardivel, Jean-Jacques Risso, Nicolas Vallee.


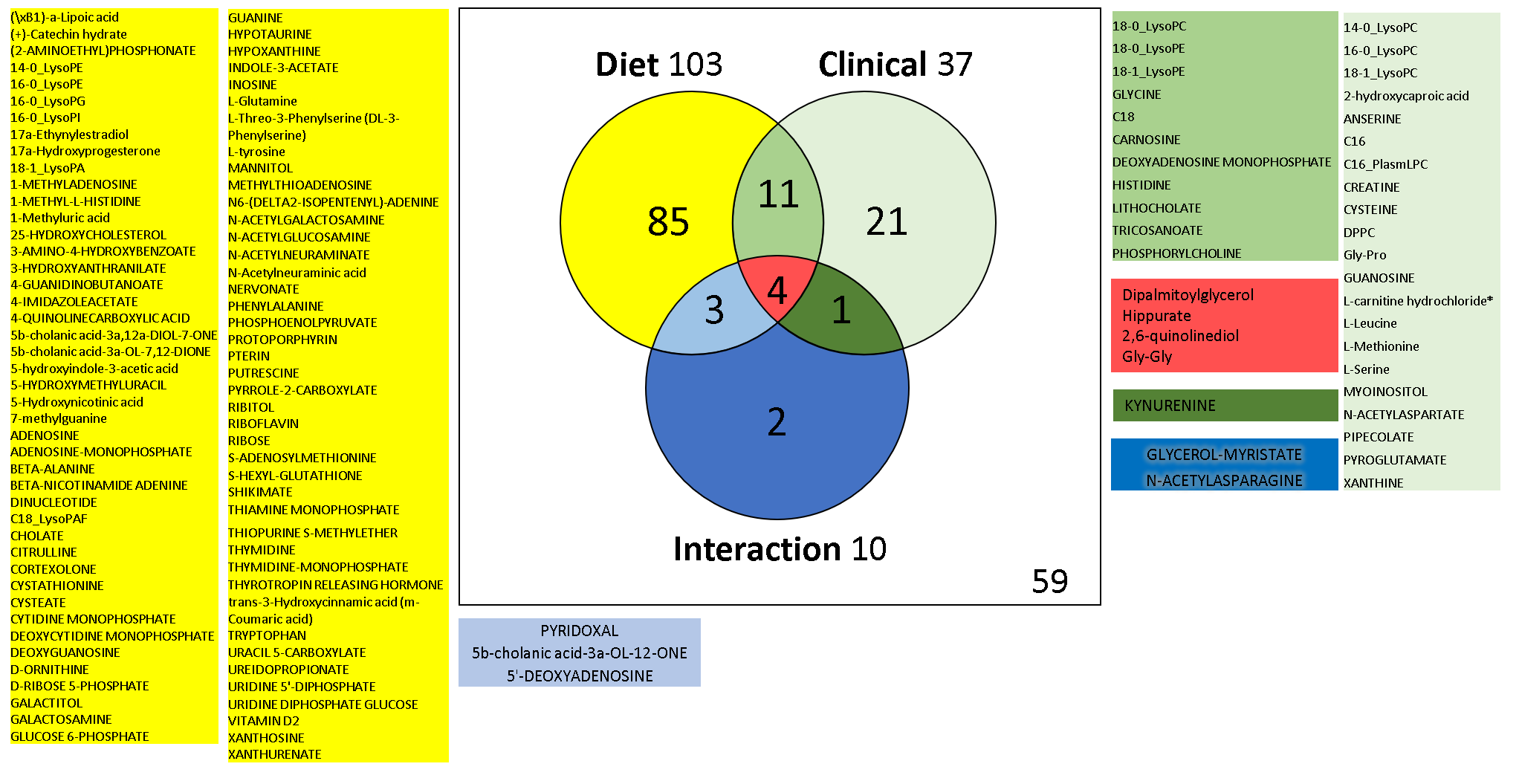
**Supp Data 1** Details of fecal metabolites altered by clinical status or diet. 185 compounds have been analyzed.

|  | **Clinic** | |
| --- | --- | --- |
| **Metabolite** | **p-value** | **Fold Change NoDCS / DCS** |
| C18 | < 0,0001 | 0.29 |
| PIPECOLATE | < 0,0001 | 0.41 |
| 14-0_LysoPC | < 0,0001 | 0.25 |
| 18-1_LysoPC | 0.000 | 0.35 |
| DPPC | 0.000 | 0.24 |
| L-carnitine hydrochloride | 0.000 | 0.49 |
| 18-0_LysoPC | 0.000 | 0.47 |
| Gly-Gly | 0.000 | 0.54 |
| CREATINE | 0.000 | 0.20 |
| 18-1_LysoPE | 0.001 | 0.54 |
| Gly-Pro | 0.001 | 0.52 |
| 16-0_LysoPC | 0.001 | 0.42 |
| C16_PlasmLPC | 0.001 | 0.28 |
| C16 | 0.001 | 0.26 |
| 18-0_LysoPE | 0.002 | 0.37 |
| MYOINOSITOL | 0.003 | 0.44 |
| HIPPURATE | 0.004 | 0.38 |
| CARNOSINE | 0.007 | 0.55 |
| XANTHINE | 0.007 | 0.66 |
| GLYCINE | 0.007 | 0.68 |
| L-Leucine | 0.008 | 0.64 |
| 2.6-quinolinediol | 0.009 | 0.41 |
| PYROGLUTAMATE | 0.011 | 0.70 |
| CYSTEINE | 0.013 | 0.44 |
| GUANOSINE | 0.014 | 0.71 |
| L-Serine | 0.016 | 0.78 |
| KYNURENINE | 0.027 | 0.73 |
| PHOSPHORYLCHOLINE | 0.027 | 0.38 |
| DIPALMITOYLGLYCEROL | 0.028 | 0.69 |
| 2-hydroxycaproic acid | 0.028 | 3.88 |
| ANSERINE | 0.033 | 0.42 |
| N-ACETYLASPARTATE | 0.033 | 0.70 |
| TRICOSANOATE | 0.035 | 0.71 |
| HISTIDINE | 0.035 | 0.82 |
| LITHOCHOLATE | 0.037 | 1.80 |
| DEOXYADENOSINE MONOPHOSPHATE | 0.043 | 0.63 |
| L-Methionine | 0.047 | 0.82 |
| THYMINE | 0.053 | 1.60 |
| 16-0_LysoPG | 0.053 | 0.67 |
| glycocholic acid hydrate | 0.056 | 0.86 |
| THYMIDINE-MONOPHOSPHATE | 0.056 | 0.60 |
| ALLANTOIN | 0.063 | 0.38 |
| DEOXYCYTIDINE MONOPHOSPHATE | 0.074 | 0.67 |
| VITAMIN D2 | 0.077 | 0.63 |
| L-tyrosine | 0.077 | 0.82 |
| ASPARTATE | 0.077 | 1.19 |
| THIAMINE MONOPHOSPHATE | 0.084 | 1.56 |
| 5-HYDROXYMETHYLURACIL | 0.089 | 1.76 |
| UREIDOPROPIONATE | 0.090 | 0.69 |
| PHENYLALANINE | 0.096 | 0.75 |
| 16-0_LysoPS | 0.096 | 2.74 |
| GALACTITOL | 0.098 | 0.83 |
| 1-METHYLADENOSINE | 0.102 | 0.78 |
| RIBITOL | 0.103 | 1.87 |
| AGMATINE SULFATE | 0.105 | 0.20 |
| GALACTOSAMINE | 0.108 | 0.79 |
| N-Acetylneuraminic acid | 0.108 | 0.70 |
| BIOTIN | 0.113 | 0.73 |
| 5'-DEOXYADENOSINE | 0.117 | 1.72 |
| XANTHOSINE | 0.121 | 0.61 |
| 16-0_LysoPE | 0.128 | 0.76 |
| PUTRESCINE | 0.131 | 0.75 |
| L-Asparagine | 0.132 | 1.19 |
| CYTIDINE MONOPHOSPHATE | 0.138 | 0.66 |
| HYPOTAURINE | 0.152 | 1.72 |
| SPHINGANINE | 0.153 | 0.81 |
| 4-IMIDAZOLEACETATE | 0.154 | 1.72 |
| INOSINE | 0.157 | 0.73 |
| CORTEXOLONE | 0.162 | 1.68 |
| URIDINE | 0.163 | 0.71 |
| CADAVERINE | 0.167 | 0.47 |
| taurocholic acid (iso1) | 0.170 | 1.82 |
| THYROTROPIN RELEASING HORMONE | 0.180 | 0.55 |
| GALACTOSE | 0.184 | 0.55 |
| Pro-Leu | 0.186 | 0.58 |
| TRYPTAMINE | 0.188 | 0.57 |
| MALTOSE | 0.191 | 0.36 |
| ALPHA-D-GLUCOSE | 0.197 | 0.64 |
| HYPOXANTHINE | 0.200 | 0.81 |
| 5-Hydroxynicotinic acid | 0.200 | 0.76 |
| URIDINE MONOPHOSPHATE | 0.200 | 0.68 |
| 18-1_LysoPS | 0.202 | 1.89 |
| CITRULLINE | 0.207 | 0.91 |
| 17a-Ethynylestradiol | 0.210 | 1.12 |
| 5-hydroxyindole-3-acetic acid | 0.216 | 1.26 |
| L-Glutamine | 0.217 | 0.72 |
| 4 carbamyl-1-methylpyridinium | 0.219 | 0.66 |
| CYSTATHIONINE | 0.221 | 0.63 |
| HOMOSERINE | 0.222 | 0.74 |
| pregnenolone sulfate | 0.222 | 0.51 |
| N-ALPHA-ACETYLLYSINE | 0.228 | 0.79 |
| GLYCEROL 3-PHOSPHATE | 0.232 | 0.28 |
| GLUCOSE 6-PHOSPHATE | 0.242 | 0.74 |
| D-ORNITHINE | 0.242 | 0.83 |
| DEOXYCYTIDINE | 0.253 | 0.85 |
| CORTISOL 21-ACETATE | 0.262 | 1.15 |
| LACTOSE | 0.270 | 0.23 |
| 14-0_LysoPE | 0.273 | 0.76 |
| BETA-ALANINE | 0.273 | 0.83 |
| STACHYOSE | 0.275 | 0.34 |
| URIDINE DIPHOSPHATE-N-ACETYLGLUCOSAMINE | 0.278 | 0.72 |
| CELLOBIOSE | 0.284 | 0.38 |
| PYRROLE-2-CARBOXYLATE | 0.285 | 0.72 |
| D-Raffinose (D-(+)-Raffinose pentahydrate ) | 0.291 | 0.41 |
| TRYPTOPHAN | 0.295 | 0.88 |
| SORBOSE | 0.308 | 1.20 |
| N-ACETYLNEURAMINATE | 0.310 | 0.81 |
| 2-DEOXY-D-GLUCOSE | 0.312 | 2.09 |
| ASPARAGINE | 0.314 | 0.82 |
| 16:0_LysoPA | 0.316 | 0.69 |
| 5b-cholanic acid-3a-OL-12-ONE | 0.316 | 1.38 |
| URIDINE DIPHOSPHATE GLUCOSE | 0.321 | 0.60 |
| INDOLE-3-ACETATE | 0.324 | 1.17 |
| BILIVERDIN | 0.327 | 0.70 |
| D-RIBOSE 5-PHOSPHATE | 0.330 | 0.81 |
| S-HEXYL-GLUTATHIONE | 0.335 | 0.81 |
| THYMIDINE | 0.338 | 0.87 |
| NERVONATE | 0.342 | 0.85 |
| SACCHARATE | 0.360 | 0.75 |
| ADENOSINE | 0.372 | 0.88 |
| 17a-Hydroxyprogesterone | 0.374 | 0.90 |
| N-Acetyl-D-sphingosine | 0.376 | 0.82 |
| CORTISOL | 0.381 | 0.78 |
| MANNITOL | 0.386 | 1.19 |
| GLUTAMATE | 0.402 | 1.10 |
| (+)-Catechin hydrate | 0.402 | 0.76 |
| ADENOSINE-MONOPHOSPHATE | 0.409 | 0.86 |
| ARGININE | 0.418 | 0.86 |
| 3-HYDROXYANTHRANILATE | 0.418 | 0.87 |
| 3-AMINO-4-HYDROXYBENZOATE | 0.424 | 0.86 |
| RIBOSE | 0.427 | 0.91 |
| L-Threo-3-Phenylserine (DL-3-Phenylserine) | 0.436 | 0.90 |
| 4-GUANIDINOBUTANOATE | 0.440 | 1.21 |
| 4-QUINOLINECARBOXYLIC ACID | 0.446 | 1.05 |
| DEOXYGUANOSINE-MONOPHOSPHATE | 0.452 | 1.13 |
| THIOPURINE S-METHYLETHER | 0.458 | 1.37 |
| GUANINE | 0.465 | 0.85 |
| BETA-NICOTINAMIDE ADENINE DINUCLEOTIDE | 0.481 | 0.84 |
| CHOLATE | 0.489 | 1.08 |
| N-ACETYLASPARAGINE | 0.491 | 0.84 |
| PROTOPORPHYRIN | 0.495 | 1.07 |
| (\xB1)-a-Lipoic acid | 0.513 | 1.33 |
| S-ADENOSYLMETHIONINE | 0.516 | 1.11 |
| 16-0_LysoPI | 0.529 | 0.82 |
| MELATONIN | 0.535 | 1.15 |
| DEOXYADENOSINE | 0.539 | 0.84 |
| 5b-cholanic acid-3-ONE | 0.547 | 0.93 |
| SHIKIMATE | 0.553 | 0.91 |
| QUINATE | 0.555 | 0.90 |
| URIDINE 5'-DIPHOSPHATE | 0.564 | 0.75 |
| C18_LysoPAF | 0.584 | 1.03 |
| GLUCOSAMINE 6-SULFATE | 0.593 | 1.14 |
| PANTOTHENATE | 0.596 | 1.10 |
| OXOGLUTARATE | 0.609 | 1.12 |
| 1-Methyluric acid | 0.617 | 0.87 |
| LYSINE | 0.635 | 0.94 |
| CYTIDINE | 0.640 | 0.92 |
| METHYLTHIOADENOSINE | 0.655 | 1.06 |
| (2-AMINOETHYL)PHOSPHONATE | 0.671 | 1.12 |
| GLUCURONATE | 0.672 | 1.08 |
| N-ACETYLGLUCOSAMINE | 0.678 | 0.95 |
| 1-METHYL-L-HISTIDINE | 0.682 | 0.96 |
| LYXOSE | 0.688 | 1.09 |
| 25-HYDROXYCHOLESTEROL | 0.705 | 1.05 |
| PHOSPHOENOLPYRUVATE | 0.732 | 0.85 |
| GLYCEROL-MYRISTATE | 0.735 | 1.08 |
| N6-(DELTA2-ISOPENTENYL)-ADENINE | 0.742 | 0.97 |
| CYSTEATE | 0.754 | 1.06 |
| 5b-cholanic acid-3a.12a-DIOL-7-ONE | 0.780 | 0.81 |
| CHENODEOXYCHOLATE | 0.798 | 1.06 |
| DEOXYGUANOSINE | 0.800 | 0.94 |
| 3-b-Hydroxyandrost-5-en-17-one (Dehydroepiandrosterone) | 0.805 | 1.02 |
| RIBOFLAVIN | 0.808 | 0.97 |
| 18-1_LysoPA | 0.815 | 1.08 |
| URACIL 5-CARBOXYLATE | 0.850 | 0.96 |
| 5b-cholanic acid-3a-OL-7.12-DIONE | 0.887 | 1.01 |
| RHAMNOSE | 0.889 | 1.02 |
| 7-methylguanine | 0.905 | 0.98 |
| PTERIN | 0.911 | 0.98 |
| DEOXYCHOLATE | 0.925 | 0.98 |
| PYRIDOXAL | 0.936 | 0.99 |
| ARABINOSE | 0.944 | 1.02 |
| N-ACETYLGALACTOSAMINE | 0.945 | 1.01 |
| XANTHURENATE | 0.959 | 1.00 |
| trans-3-Hydroxycinnamic acid (m-Coumaric acid) | 0.967 | 1.01 |

|  | **Diet** | |
| --- | --- | --- |
| **Metabolite** | **p-value** | **Fold Change CORN / SOY** |
| URACIL 5-CARBOXYLATE | 0.000 | 2.54 |
| 18-1_LysoPE | 0.000 | 0.50 |
| N-Acetylneuraminic acid | 0.000 | 2.62 |
| HYPOXANTHINE | 0.000 | 0.52 |
| 1-Methyluric acid | 0.000 | 0.31 |
| BETA-NICOTINAMIDE ADENINE DINUCLEOTIDE | 0.000 | 0.34 |
| UREIDOPROPIONATE | 0.000 | 0.42 |
| ADENOSINE | 0.000 | 0.56 |
| C18_LysoPAF | 0.000 | 1.26 |
| THIOPURINE S-METHYLETHER | 0.001 | 0.13 |
| THYMIDINE | 0.001 | 0.59 |
| (\xB1)-a-Lipoic acid | 0.001 | 0.13 |
| CYSTATHIONINE | 0.001 | 0.23 |
| GLYCINE | 0.001 | 0.63 |
| PHOSPHORYLCHOLINE | 0.001 | 0.20 |
| 5b-cholanic acid-3a-OL-12-ONE | 0.001 | 3.18 |
| BETA-ALANINE | 0.002 | 0.56 |
| D-ORNITHINE | 0.003 | 0.61 |
| XANTHOSINE | 0.003 | 0.35 |
| DEOXYGUANOSINE | 0.003 | 2.36 |
| PTERIN | 0.003 | 0.49 |
| 18-0_LysoPE | 0.003 | 0.38 |
| CYTIDINE MONOPHOSPHATE | 0.004 | 0.43 |
| INOSINE | 0.005 | 0.53 |
| THYMIDINE-MONOPHOSPHATE | 0.006 | 0.47 |
| L-tyrosine | 0.006 | 0.73 |
| HISTIDINE | 0.007 | 0.77 |
| N-ACETYLGLUCOSAMINE | 0.007 | 1.38 |
| RIBITOL | 0.009 | 0.34 |
| GLUCOSE 6-PHOSPHATE | 0.010 | 0.49 |
| DEOXYCYTIDINE MONOPHOSPHATE | 0.011 | 0.55 |
| 1-METHYLADENOSINE | 0.011 | 0.67 |
| GALACTITOL | 0.013 | 0.76 |
| 4-IMIDAZOLEACETATE | 0.013 | 2.77 |
| HYPOTAURINE | 0.015 | 2.67 |
| RIBOFLAVIN | 0.015 | 0.69 |
| URIDINE 5'-DIPHOSPHATE | 0.016 | 4.27 |
| (2-AMINOETHYL)PHOSPHONATE | 0.016 | 0.50 |
| 1-METHYL-L-HISTIDINE | 0.016 | 1.32 |
| 17a-Hydroxyprogesterone | 0.017 | 0.74 |
| 18-1_LysoPA | 0.018 | 0.44 |
| 5-Hydroxynicotinic acid | 0.018 | 0.59 |
| GUANINE | 0.019 | 1.70 |
| THIAMINE MONOPHOSPHATE | 0.019 | 0.54 |
| URIDINE DIPHOSPHATE GLUCOSE | 0.020 | 3.93 |
| 5-HYDROXYMETHYLURACIL | 0.022 | 0.45 |
| PHENYLALANINE | 0.024 | 0.67 |
| 4-GUANIDINOBUTANOATE | 0.026 | 0.56 |
| CITRULLINE | 0.032 | 0.86 |
| 16-0_LysoPI | 0.033 | 0.48 |
| 18-0_LysoPC | 0.040 | 0.67 |
| PUTRESCINE | 0.040 | 0.67 |
| C18 | 0.043 | 0.60 |
| PHOSPHOENOLPYRUVATE | 0.047 | 0.35 |
| 5b-cholanic acid-3a.12a-DIOL-7-ONE | 0.048 | 7.23 |
| (+)-Catechin hydrate | 0.049 | 0.50 |
| GUANOSINE | 0.053 | 0.77 |
| ANSERINE | 0.056 | 0.46 |
| BILIVERDIN | 0.058 | 0.49 |
| 3-b-Hydroxyandrost-5-en-17-one (Dehydroepiandrosterone) | 0.077 | 0.88 |
| LYSINE | 0.079 | 0.80 |
| pregnenolone sulfate | 0.082 | 2.71 |
| MELATONIN | 0.087 | 0.68 |
| 16:0_LysoPA | 0.092 | 0.53 |
| QUINATE | 0.094 | 0.73 |
| THYMINE | 0.098 | 1.49 |
| URIDINE | 0.103 | 0.67 |
| XANTHINE | 0.106 | 0.78 |
| MYOINOSITOL | 0.115 | 1.50 |
| 2-DEOXY-D-GLUCOSE | 0.139 | 0.32 |
| STACHYOSE | 0.143 | 4.84 |
| N-ACETYLASPARTATE | 0.165 | 1.25 |
| DEOXYGUANOSINE-MONOPHOSPHATE | 0.174 | 0.80 |
| glycocholic acid hydrate | 0.183 | 1.11 |
| L-Leucine | 0.187 | 0.81 |
| N-Acetyl-D-sphingosine | 0.196 | 0.74 |
| 14-0_LysoPC | 0.196 | 0.69 |
| L-Methionine | 0.207 | 1.13 |
| ASPARTATE | 0.213 | 0.89 |
| URIDINE DIPHOSPHATE-N-ACETYLGLUCOSAMINE | 0.224 | 0.69 |
| CHENODEOXYCHOLATE | 0.241 | 1.29 |
| L-Asparagine | 0.243 | 0.88 |
| HOMOSERINE | 0.265 | 0.76 |
| ARGININE | 0.276 | 0.81 |
| GLUCURONATE | 0.286 | 0.82 |
| OXOGLUTARATE | 0.293 | 1.26 |
| BIOTIN | 0.299 | 0.81 |
| L-Serine | 0.307 | 0.90 |
| RHAMNOSE | 0.310 | 0.85 |
| CYSTEINE | 0.323 | 1.35 |
| 16-0_LysoPS | 0.368 | 1.67 |
| PANTOTHENATE | 0.386 | 0.85 |
| DPPC | 0.387 | 0.77 |
| ALLANTOIN | 0.396 | 1.51 |
| CYTIDINE | 0.396 | 0.87 |
| D-Raffinose (D-(+)-Raffinose pentahydrate ) | 0.446 | 1.87 |
| PYROGLUTAMATE | 0.447 | 0.90 |
| 5b-cholanic acid-3-ONE | 0.466 | 1.10 |
| CELLOBIOSE | 0.481 | 1.84 |
| AGMATINE SULFATE | 0.487 | 1.80 |
| taurocholic acid (iso1) | 0.494 | 1.34 |
| ALPHA-D-GLUCOSE | 0.495 | 1.26 |
| DEOXYCYTIDINE | 0.496 | 0.91 |
| ARABINOSE | 0.512 | 0.86 |
| CADAVERINE | 0.526 | 1.40 |
| LACTOSE | 0.533 | 2.08 |
| SORBOSE | 0.534 | 0.90 |
| CORTISOL | 0.534 | 0.84 |
| SPHINGANINE | 0.573 | 1.09 |
| TRYPTAMINE | 0.577 | 0.79 |
| 18-1_LysoPS | 0.583 | 1.30 |
| GLYCEROL 3-PHOSPHATE | 0.586 | 1.68 |
| C16_PlasmLPC | 0.617 | 1.18 |
| 16-0_LysoPC | 0.622 | 1.12 |
| CORTISOL 21-ACETATE | 0.626 | 1.06 |
| N-ALPHA-ACETYLLYSINE | 0.628 | 1.10 |
| LYXOSE | 0.666 | 1.09 |
| 4 carbamyl-1-methylpyridinium | 0.672 | 1.15 |
| MALTOSE | 0.680 | 1.34 |
| CREATINE | 0.696 | 1.15 |
| KYNURENINE | 0.704 | 0.95 |
| 18-1_LysoPC | 0.720 | 0.92 |
| URIDINE MONOPHOSPHATE | 0.738 | 0.91 |
| SACCHARATE | 0.768 | 0.91 |
| C16 | 0.801 | 1.09 |
| PIPECOLATE | 0.830 | 1.04 |
| GLUTAMATE | 0.851 | 1.02 |
| DEOXYADENOSINE | 0.859 | 1.05 |
| GALACTOSE | 0.892 | 0.94 |
| GLUCOSAMINE 6-SULFATE | 0.923 | 0.98 |
| 2-hydroxycaproic acid | 0.938 | 1.04 |
| ASPARAGINE | 0.940 | 1.01 |
| DEOXYCHOLATE | 0.942 | 1.01 |
| N-ACETYLASPARAGINE | 0.943 | 1.02 |
| L-carnitine hydrochloride | 0.948 | 1.01 |
| Pro-Leu | 0.952 | 1.02 |
| GLYCEROL-MYRISTATE | 0.973 | 1.01 |
| Gly-Pro | 0.976 | 1.01 |
| 14-0_LysoPE | < 0,0001 | 0.20 |
| 16-0_LysoPE | < 0,0001 | 0.30 |
| 16-0_LysoPG | < 0,0001 | 0.40 |
| 17a-Ethynylestradiol | < 0,0001 | 2.53 |
| 2.6-quinolinediol | < 0,0001 | 332.87 |
| 25-HYDROXYCHOLESTEROL | < 0,0001 | 1.97 |
| 3-AMINO-4-HYDROXYBENZOATE | < 0,0001 | 12.84 |
| 3-HYDROXYANTHRANILATE | < 0,0001 | 15.25 |
| 4-QUINOLINECARBOXYLIC ACID | < 0,0001 | 0.22 |
| 5b-cholanic acid-3a-OL-7.12-DIONE | < 0,0001 | 1.80 |
| 5'-DEOXYADENOSINE | < 0,0001 | 0.09 |
| 5-hydroxyindole-3-acetic acid | < 0,0001 | 6.98 |
| 7-methylguanine | < 0,0001 | 0.17 |
| ADENOSINE-MONOPHOSPHATE | < 0,0001 | 3.27 |
| CARNOSINE | < 0,0001 | 0.36 |
| CHOLATE | < 0,0001 | 3.23 |
| CORTEXOLONE | < 0,0001 | 17.82 |
| CYSTEATE | < 0,0001 | 0.24 |
| DEOXYADENOSINE MONOPHOSPHATE | < 0,0001 | 0.33 |
| DIPALMITOYLGLYCEROL | < 0,0001 | 0.00 |
| D-RIBOSE 5-PHOSPHATE | < 0,0001 | 0.37 |
| GALACTOSAMINE | < 0,0001 | 0.43 |
| Gly-Gly | < 0,0001 | 0.38 |
| HIPPURATE | < 0,0001 | 249.94 |
| INDOLE-3-ACETATE | < 0,0001 | 33.15 |
| L-Glutamine | < 0,0001 | 0.29 |
| LITHOCHOLATE | < 0,0001 | 4.20 |
| L-Threo-3-Phenylserine (DL-3-Phenylserine) | < 0,0001 | 3.51 |
| MANNITOL | < 0,0001 | 0.36 |
| METHYLTHIOADENOSINE | < 0,0001 | 0.24 |
| N6-(DELTA2-ISOPENTENYL)-ADENINE | < 0,0001 | 0.36 |
| N-ACETYLGALACTOSAMINE | < 0,0001 | 1.86 |
| N-ACETYLNEURAMINATE | < 0,0001 | 3.86 |
| NERVONATE | < 0,0001 | 0.27 |
| PROTOPORPHYRIN | < 0,0001 | 2.96 |
| PYRIDOXAL | < 0,0001 | 1.95 |
| PYRROLE-2-CARBOXYLATE | < 0,0001 | 28.99 |
| RIBOSE | < 0,0001 | 0.29 |
| S-ADENOSYLMETHIONINE | < 0,0001 | 0.17 |
| S-HEXYL-GLUTATHIONE | < 0,0001 | 0.15 |
| SHIKIMATE | < 0,0001 | 0.31 |
| THYROTROPIN RELEASING HORMONE | < 0,0001 | 0.05 |
| trans-3-Hydroxycinnamic acid (m-Coumaric acid) | < 0,0001 | 0.00 |
| TRICOSANOATE | < 0,0001 | 0.39 |
| TRYPTOPHAN | < 0,0001 | 0.53 |
| VITAMIN D2 | < 0,0001 | 0.28 |
| XANTHURENATE | < 0,0001 | 0.13 |

Supp Data 2 Use of the ChemRICH database (Barupal and Fiehn, 2017) allows the easy grouping of significantly altered metabolites depending on their chemical similarity (Figure below). It is therefore observed that the soy diet has a tendency to increase the quantity of amino-acids and saturated lysophospholipids and to reduce that of cholic acids in the feces.


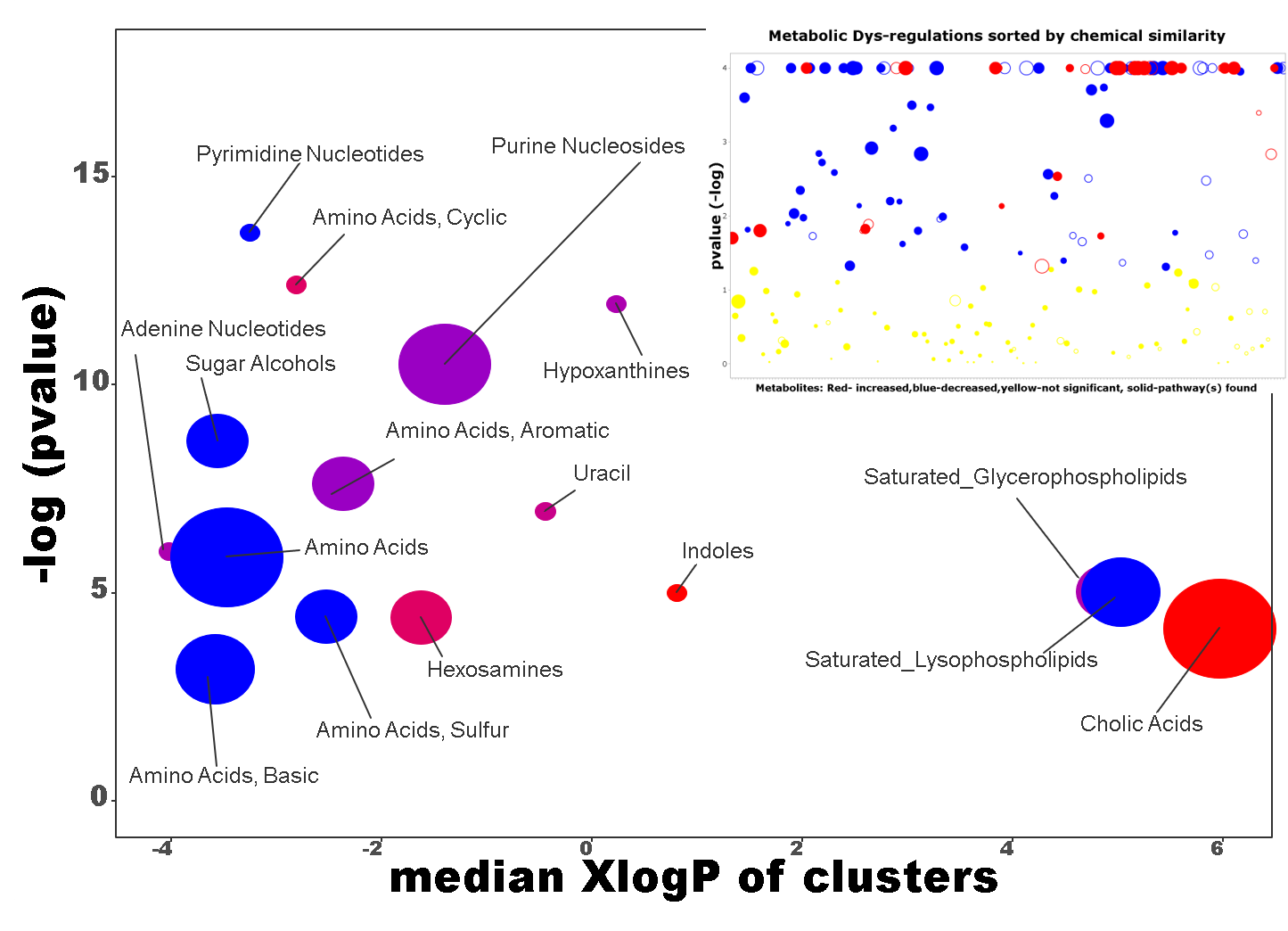


**Figure 11** Graph of ChemRICH enrichment statistics for altered metabolites as a function of diet. Each circle represents a group of significantly modified metabolites. These groups are constituted from the chemical similarities shown by a Tanimoto hierarchical map (not shown) accessible in the ChemRICH program. The enrichment p values are given by the Kolmogorov-Smirnov test. The size of the circles represents the total number of metabolites in each group. The circle color scale shows the increased (red) or decreased (blue) proportion of compounds in rats fed on maize compared with those fed with soy. The purple circles have both an increase and a decrease in metabolites. For example, more cholic acids are found in the feces of MAIZE rats compared with SOY rats. Insert: Volcano plot showing the metabolic dysregulation as a function of the diet of the rats, specifying for each compound whether the metabolic pathway is recognized by ChemRICH.
